# High-molecular-weight hyaluronic acid attenuated matrix metalloproteinase-1 and -3 expression via CD44 in tendinopathy

**DOI:** 10.1038/srep40840

**Published:** 2017-01-16

**Authors:** Po-Ting Wu, Li-Chieh Kuo, Fong-Chin Su, Shih-Yao Chen, Tai-I Hsu, Chung-Yi Li, Kuen-Jer Tsai, I-Ming Jou

**Affiliations:** 1 Department of Orthopedics, College of Medicine, National Cheng Kung University, Tainan, Taiwan; 2 Department of Orthopedics, National Cheng Kung University Hospital, College of Medicine, National Cheng Kung University, Tainan, Taiwan; 3 Department of Biomedical Engineering, National Cheng Kung University, Tainan, Taiwan; 4 Medical Device Innovation Center, National Cheng Kung University, Tainan, Taiwan; 5 Department of Occupational Therapy, National Cheng Kung University, Tainan, Taiwan; 6 Department of Internal Medicine, College of Medicine, National Cheng Kung University, Tainan, Taiwan; 7 Department of Orthopedics, E-DA Hospital, Kaohsiung, Taiwan; 8 Department of Public Health, College of Medicine, National Cheng Kung University, Tainan, Taiwan; 9 Institute of Clinical Medicine, College of Medicine, National Cheng Kung University, Tainan, Taiwan; 10 Center of Clinical Medicine, National Cheng Kung University Hospital, College of Medicine, National Cheng Kung University, Tainan, Taiwan

## Abstract

Evidence indicates that hyaluronic acid (HA) mitigates tendinopathy, but the effect of molecular weight is unclear. We investigated the effects of different concentrations and different molecular weights of HA (350 kDa, 1500 kDa, and 3000 kDa) on matrix metalloproteinase (MMP)-1 and -3 expression in IL-1β-stimulated rat tenocytes, and on their dynamic expression in peritendinous effusion from patients with long head of biceps (LHB) tendinopathy after high-molecular-weight (HMW)-HA treatments. Reverse transcription PCR, real-time PCR, and ELISA were used to determine MMP-1 and -3expression. Because CD44 was clearly expressed in the plasma membranes of cultured tenocytes, OX-50, a CD44 antagonist, was used to inhibit CD44 to evaluate the HA mechanism. HA (3000 kDa) significantly (p < 0.001) downregulated the mRNA and protein expression of MMP-1 and -3 in IL-1β-stimulated tenocytes. Its attenuating effects were dose-dependent (p < 0.01). In OX-50-pretreated cells, the mRNA expression of CD44 was not significantly altered, but the mRNA expression of MMP-1 and -3 was significantly upregulated. Visual analogue scale scores were significantly lower, and MMP-1 and -3 expression was significantly (p < 0.05) lower one month posttreatment. HMW-HA attenuated tendinopathy by downregulating MMP-1 and -3 expression. Inhibiting CD44 blocked the effects of HMW-HA.

Tendon overuse injury is the most common soft tissue injury and is claimed to account for 30–50% of all sports-related injuries^1^. Nonsurgical management is the first choice of treatment for most cases^2^,^3^,^4^. Emerging clinical evidence shows that significant harm to tendon tissue is associated with local corticosteroid injections^5^. Therefore, these significant negative effects have contributed to the greater use of other locally injectable tendinopathy therapeutics, such as lauromacrogol (polidocanol), platelet-rich plasma, botulinum toxin, proteinases, and hyaluronic acid (HA).

HA, a large glycosaminoglycan, regulates physiological processes in almost all tissue^6^. CD44 is a primary HA receptor with multiple physiological and pathological functions^7^,^8^. Other studies^9^,^10^ have shown that HA modulates inflammation based on its different degrees of polymerization (i.e., molecular weight). High-molecular-weight (HMW)-HA reduces inflammation in experimental arthritis^11^ and in interleukin (IL)-1β-stimulated synovial fibroblasts^12^. Despite evidence from clinical trials^13^,^14^,^15^,^16^ and animal studies^17^,^18^,^19^ that HA mitigates tendinopathy, the molecular-weight effect of HA on tenocytes in tendinopathy is still unclear.

Because IL-1β, a proinflammatory cytokine, regulates inflammatory mediators such as COX-2 and matrix metalloproteinases(MMPs)^20^,^21^, it is one of the initiators of tendinopathy. MMPs degrade the extracellular matrix (ECM) and mediate the development of tendinopathy and even tendon rupture^22^. Others^21^,^23^ have reported significantly elevated MMP-1 and -3 expression after IL-1β stimulation in human tendon cells and have made IL-1β a candidate for an *in vitro* tendinopathy model. In contrast, HMW-HA has downregulated MMP expression *in vivo*^24^ and *in vitro* after IL-1β stimulation in arthritis studies^24^,^25^. Therefore, we hypothesized that HMW-HA downregulates MMP-1 and -3 expression by stimulating CD44 receptor in tendinopathy. We investigated the effects of three molecular weights and three concentrations of HA on the expression of MMP-1 and -3 in IL-1β-stimulated tenocytes from rat Achilles tendons. Long head of biceps (LHB) tendinopathy is a recognized cause of shoulder pain^26^,^27^ and is usually associated with other shoulder abnormalities, including rotator cuff tears^27^,^28^. Because of the high incidence of LHB tendinopathy in chronic shoulder pain^29^, the treatment has gained more and more attention. Bicipital peritendinous effusion has been reported strong association with LHB tendinopathy^28^ and is the result of the excess synovial secretion by the tendon sheath^30^. Therefore, we also evaluated the dynamic MMP-1 and -3 expression in peritendinous effusion from patients with LHB tendinopathy after HMW-HA treatments. Tenocytes were also pretreated with OX-50 (anti-CD44 antibody) to evaluate the mechanism of action of HA.

## Results

### Identifying tenocytes from primary culture

**Figure 1:**
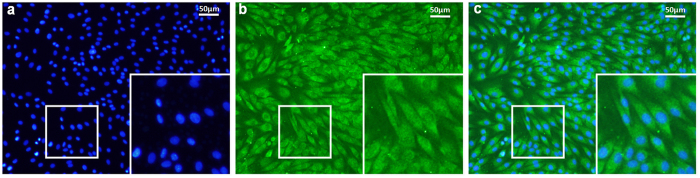
Immunofluorescent staining of tenomodulin in the primary cultured tenocytes from the rats’ Achilles tendons. The cultured cells were co-stained with (**a**) DAPI (blue) and (**b**) anti-tenomodulin (green). (**c**) All cells showed tenocyte characteristics, including tenomodulin expression and a typical spindle shape. The bar is 50 μm.

The vast majority of cultured tendon cells showed a clear elongated fibroblastic appearance under the microscope. Every cell was immunopositive for tenomodulin, the tenocyte marker (Fig. 1), which confirmed that the primary cultured cells were tenocytes.

### Effects of HA on tenocyte proliferation and viability

**Figure 2:**
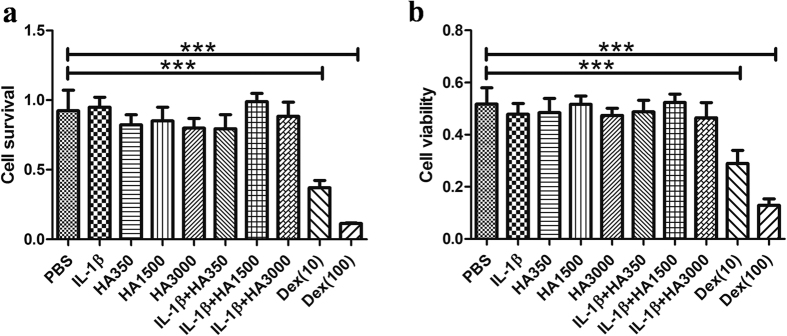
Tenocyteswere treated for 24 h with IL-1β (10 ng/mL), HA350 (HA350 kDa), HA1500 (HA1500 kDa), HA3000 (HA3000 kDa), various combinations of IL-1β and HA (2.5 mg/mL), and Dex (dexamethasone)(10 nM or 100 nM). Cell proliferation and viability were assessed using PrestoBlue (**a**) and MTT (**b**) assays, respectively. ***p < 0.001 compared with Controls (PBS-only).

After they had been treated with IL-1β, or with HA of various molecular weights, or with both for 24 h, there were no obvious morphologically changed tenocytes that maintained a bipolar and spindle shape. PrestoBlue and MTT assays showed no differences between the proliferation or viability of HA-treated tenocytes and the PBS-treated Controls. However, the proliferation and viability of tenocytes treated with either 10 nM or 100 nM of Dex were significantly (p < 0.01) and dose-dependently lower than those of the PBS-treated Controls (Fig. 2).

### Effects of HA of various molecular weights on the mRNA and protein expression of MMP-1 and -3

**Figure 3:**
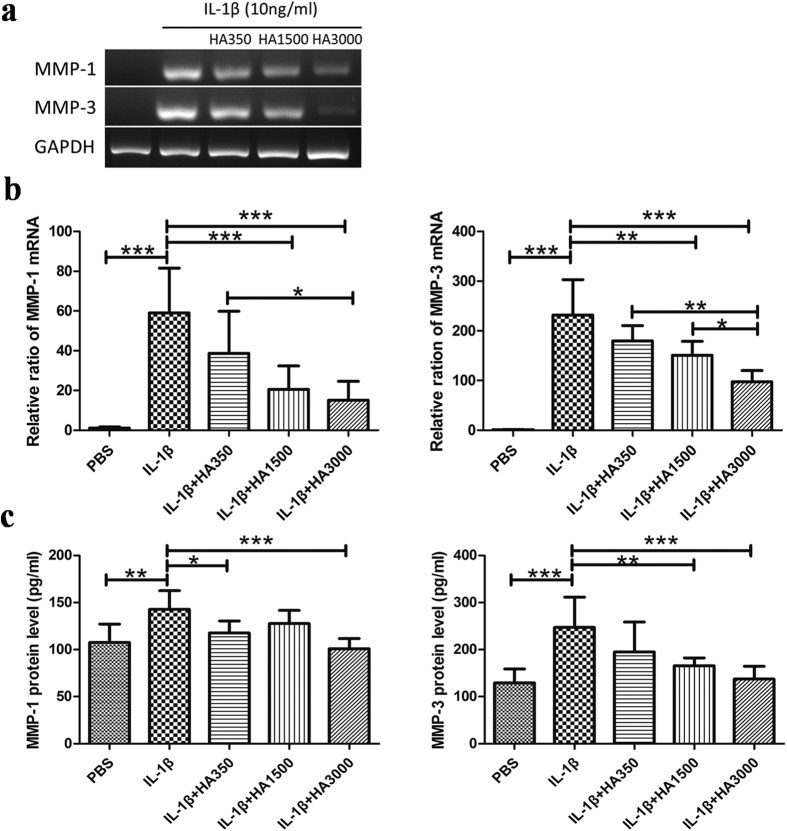
Effects of HA on the mRNA and protein expressions of MMP-1 and -3. Total RNA and total protein were isolated from the tenocytes that had been treated for 24 h with IL-1β (10 ng/mL) in the absence or presence of HA preparations of various molecular weights (2.5 mg/mL for HA350, HA1500, and HA3000). The PBS-only group was the Control group. Gene expression levels were evaluated using RT-PCR (**a**) and real-time RT-PCR (**b**). Protein levels were assessed using ELISA (**c**). Bars are mean ± SD. *p < 0.05, **p < 0.01, ***p < 0.001.

RT-PCR, real-time PCR, and ELISA showed that the protein and mRNA expression of MMP-1 and -3 was low in the PBS-treated Control but significantly overexpressed in the IL-1β-only-treated tenocytes (Fig. 3). After tenocytes had been co-treated withIL-1β with or without HA of various molecular weights, real-time PCR showed the mean relative ratios of the mRNA expression levels of MMP-1 and -3, respectively, as: 51.4 ± 20.5 and 239.5 ± 71.4 in the IL-1β-only-treated group; 38.7 ± 21.1 and 172.6 ± 37 in the HA350 group; 20.5 ± 11.8 and 138.8 ± 31.49 in the HA1500 group; and 15.1 ± 9.5 and 127.8 ± 45.83 in the HA3000 group: mRNA expression of MMP-1 and -3 was significantly (p < 0.001) lower in the HA1500 and HA3000 groups than in the IL-1β-only-treated group (Fig. 3b). ELISA showed that the mean protein expression levels of MMP-1 and -3 were: 142.9 ± 22.9 and 274.3 ± 64.2 in the IL-1β-only-treated group; 117.7 ± 11.7 and 195.0 ± 63.4 in the HA350 group; 124.7 ± 14.1 and 165.9 ± 16.5 in the HA1500 group; and 101.1 ± 10.7 and 137.4 ± 27.0 in the HA3000 group: protein expression of MMP-1 and -3 after IL-1β treatment was significantly (p < 0.001) lower only in the HA3000 group (Fig. 3c).

### Effects of various concentrations of HA on MMP-1 and -3 mRNA expression

**Figure 4:**
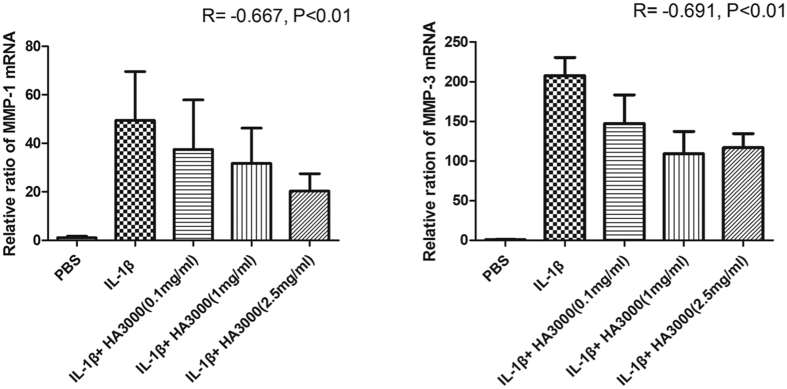
Dose effect of HA3000 on the mRNA expression of MMP-1 and -3. Total RNA was isolated from the tenocytes that had been treated for 24 h with IL-1β (10 ng/mL) in the absence or presence of HA3000 preparations of various concentrations (0.1 mg/mL, 1 mg/mL, and 2.5 mg/mL). The PBS-only group was the Control group. Gene expression levels were evaluated using real-time RT-PCR. Bars are mean ± SD.

Because MMP-1 and -3 expression was significantly downregulated in HA3000-treated cells, we used that treatment to further evaluate the effects of different concentrations onMMP-1 and -3 expression. Real-time PCR showed that the mean relative ratios of the mRNA expression levels of MMP-1 and -3 were: 44.6 ± 19 and 207.7 ± 22 in cells treated with IL-1β-only; 41.28 ± 18.4 and 147.3.6 ± 36.1 in cells treated with 0.1 mg/mL; 29.8.5 ± 12.4 and 109.24 ± 27.9 in cells treated with 1 mg/mL; and 15.2 ± 11.7 and 116.9 ± 17.5 in cells treated with 2.5 mg/mL (Fig. 4). Our results showed that mRNA expression of MMP-3 was significantly lower in cells treated with all three concentrations of HA (p < 0.05). In contrast, mRNA expression of MMP-1 was significantly (p < 0.01) lower only in 2.5 mg/mL (p < 0.05). Moreover, in either MMP-1 or -3 expression, the attenuating effects of HA were dose-dependent (MMP-1: R = −0.667, p < 0.01; MMP-3: R = −0.691, p < 0.01).

### The attenuating effects of HA were blocked in OX-50-pretreated tenocytes

**Figure 5:**
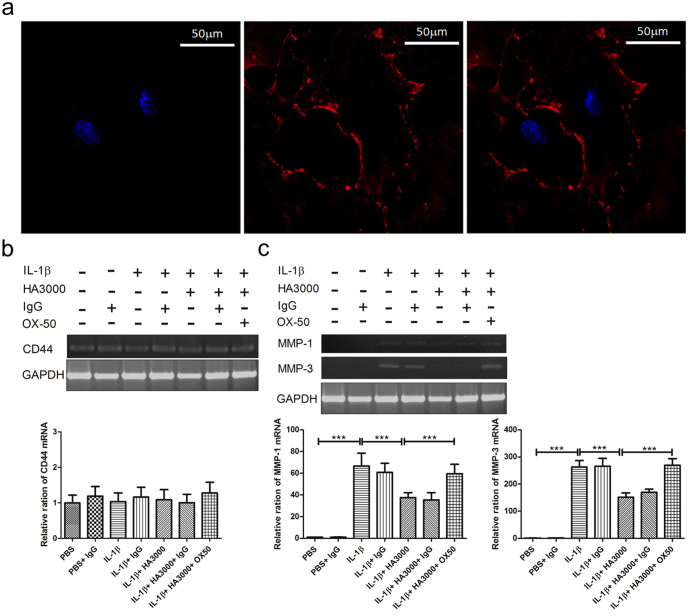
Immunofluorescent staining of CD44 in the primary cultured tenocytes. (**a**) The cultures cell were co-stained with DAPI (blue) and anti-CD44 (red). The CD44 staining was clearly localized in the plasma membranes and cell-cell junctions. The bar is 50 μm. (**b**) The mRNA expression of CD44 was not altered after various treatments. (**c**) Inhibiting the expression of CD44 blocked the attenuating effect of HA3000 on MMP-1 and -3. Tenocytes were pretreated with or without OX-50, and then treated with IL-1β-only or IL-1β + HA3000. The irrelevant rat IgG antibody was used as the OX-50 isotype control. The mRNA expression of MMP-1 and -3 was evaluated using RT-PCR and real-time RT-PCR. Bars are mean ± SD. ***p < 0.001.

CD44 is one of the major HA receptors^7^,^8^. Because CD44 was clearly expressed in the plasma membranes and cell-cell junctions of cultured tenocytes (Fig. 5a), OX-50, a CD44 antagonist, was used to inhibit CD44 expression^31^ to evaluate the mechanism of action of HA. The mRNA expression of CD44 was not significantly altered after treatments with PBS (basal Control), IL-1β, IL-1β plus HA3000, OX-50 pretreatment and IL-1β plus HA3000, and IgG (OX-50 isotype Control) (Fig. 5b). The attenuating effects of HA3000 on the mRNA expression of MMP-1 and -3 in IL-1β stimulated tenocytes were significantly (p < 0.01) reversed in tenocytes pretreated with OX-50 (Fig. 5c).

### The dynamic changes of MMPs and VAS in patients with long head of biceps tendinopathy after high-molecular-weight HA treatment

**Figure 6:**
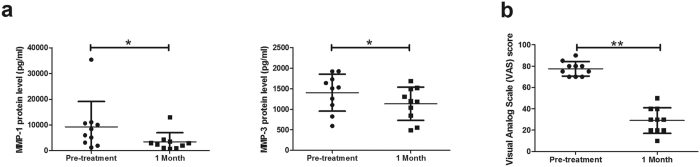
The protein expression of MMP-1 and -3 (**a**) and the VAS scale (**b**) in patients with long head of biceps (LHB) tendinopathy before and after HA treatment. Ten patients were given three weekly ultrasound-guided paratendinous injections of high-molecular-weight HA. To analyze the protein expression of MMP-1 and -3, the peritendinous effusion of LHB was collected before treatment and one month after the initial treatment. Data are expressed as mean ± SD. The differences before and after treatment were analyzed using a Wilcoxon signed rank test. *p < 0.05; 1 Month: one month after initial HA treatment.

The protein expression of MMP-1 and -3 in LHB peritendinous effusion was significantly (p < 0.05) lower one month after treatment: MMP-1 fell from 9226.9, 7115.4, and 2764.4–10677.0 (mean, median, and IQR) pg/mL to 3381.2, 2646.8, and 1047.8–3718.3 pg/mL, and MMP-3 from 1402.3, 1519.0, and 1033.4–1777.6 pg/mL to 1172.5, 1194.6, and 773.5–1539.4 pg/mL (Fig. 6a and Supplementary Table 1). Post-treatment VAS scores were also significantly (p < 0.01) lower after one month (from 77.5, 77.5, and 70.0–81.3 to 29.0, 30.0, and 20.0–40.0; Fig.6b and Supplementary Table 1).

## Discussion

This is the first study on the effects of the molecular weight and concentration of HA on the expression of MMP-1 and -3 in IL-1β-stimulated tenocytes, and on the dynamic expression of MMP-1 and -3 in peritendinous effusion after HMW-HA treatments. Unlike corticosteroid, HA did not affect the proliferation or viability of tenocytes. HA3000 significantly attenuated the mRNA and protein expression of MMP-1 and -3 in IL-1β-stimulated tenocytes. Its attenuating effects were dose-dependent (p < 0.01). CD44 was clearly expressed in the plasma membranes of cultured tenocytes. In OX-50-pretreated cells, the mRNA expression of CD44 was not altered, but the mRNA expression of MMP-1 and -3 was significantly upregulated compared with those without OX-50 pretreatment. HMW-HA also significantly attenuated MMP-1 and -3 expression in the patients with peritendinous effusion of LHB one month posttreatment.

Many molecular changes occur within an injured tendon, but the exact pathogenesis of tendinopathy is unknown. IL-1β, a proinflammatory cytokine, has been proposed as the initiator of tendinopathy because it induces inflammation, apoptosis, and ECM degradation by activating MMPs^20^. MMPs, a large family of endopeptidases, have been implicated in pain generation^32^ and tendinopathy^22^ because they cleave ECM proteins. MMP-1 and -3 are two major MMPs involved in tendinopathy. MMP-1 is one kind of collagenase which can cleave all subtypes of collagen that provide mechanical strength to tissue^33^. MMP-3, one kind of stromelysin, might be involved in Achilles tendinopathy^34^ and in the failure of normal matrix remodeling^35^. The expression levels of MMP-1 and -3 can significantly increase in tenocytes after they have been stimulated by IL-1β^21^, and they are elevated in the synovial fluid of patients with massive rotator cuff tears^36^.

We found that the *in vitro* mRNA and protein expression of both MMPs was significantly lower in tenocytes treated with HA3000. Moreover, VAS scores and MMP-1 and -3 expression were significantly lower in patients with LHB tendinopathy who had been treated with HMW-HA. These findings correspond to current clinical reports^13^,^14^,^15^,^16^ that HA is effective for treating tendinopathy. The therapeutic effects of HA might result in part from its attenuation of MMP-1 and -3 expression.

HA is known for pain control in knee osteoarthritis. Recent studies report that HA protects cartilage not only via viscosupplementation but also by suppressing the production of OA-associated cytokines such as IL-1β, IL-6, and TNF-α^37^, and of ECM-degrading proteins such as MMP-1, -3, -13^38^, and ADAMTS4^39^ in human chondrocytes. The effects of HA depend upon its molecular weight^9^,^10^. HMW-HA showed better anti-inflammation effects in experimental arthritis^11^, and IL-1β stimulated synovial fibroblasts^12^ and human osteoarthritic chondrocytes^39^. HA has also effectively mitigated tendinopathy in clinical practice^13^,^14^,^15^,^16^ and animal studies^17^,^18^,^19^. The therapeutic effects of different molecular weights of HA on tendinopathy remain unknown. Osti *et al*.^40^ reported that in human rotator cuff tendon-derived cells, various HA preparations dose-dependently stimulated the synthesis of collagen type I over 14 days, and that the preparation with the highest molecular weight upregulated the expression of collagen type I significantly more than did the other preparations. The viability and proliferation of the cells also increased for all the HA preparations utilized, but the difference was not significant compared with the control cells. Yoshida *et al*.^19^ reported that HMW-HA was effective for pain relief and for partial restoration of the patellar tendon in a rat tendinopathy model. In the present study, however, we found that regardless of their molecular weight, none of our HA preparations increased or decreased the viability or proliferation of tenocytes. In contrast, Scutt *et al*.^41^ reported that dexamethasone concentration-dependently reduced the number of cells and collagen synthesis in tenocyte cultures. Therefore, HA is not cytotoxic for tenocytes. Moreover, HA3000 more significantly attenuated the mRNA and protein expression of MMP-1 and -3 in IL-1β-stimulated tenocytes than did HA preparations of other molecular weights. Other studies have reported that HA at different concentrations significantly and dose-dependently decreased the expression levels of proinflammatory cytokines in subacromial synovial fibroblasts from patients with rotator cuff disease^42^ and of adhesion-related cytokines in glenohumeral synovial/capsular fibroblasts^43^. We too found a dose-dependent relationship that indicates that HMW-HA (HA3000) is efficacious for attenuating MMP-1 and -3 expression in IL-1β-stimulated tenocytes.

CD44, a transmembrane glycoprotein, is one of the major HA-binding proteins, and widely expressed on T cells, monocytes, granulocytes, and fibroblasts^8^. The interaction between HA and CD44 regulates development, inflammation, T cell recruitment and activation, tumor growth, and metastasis^44^. Others^42^ report that the HA-CD44 interaction reduced the expression of proinflammatory cytokine mRNAs and cyclooxygenase-2 (COX-2)/prostaglandin E2 (PGE2) production in IL-1-stimulated subacromial synovial fibroblasts, and suppressed the expression of MMP-1, -3, and -13 in IL-1β-stimulated chondrocytes^38^. In the present study, CD44 was clearly expressed in the plasma membranes and cell-cell junctions of the cultured tenocytes. Its mRNA expression was not altered after a variety of treatments. Isa *et al*.^45^ also reported that the mean fluorescence intensity of CD44 receptors in nucleus pulposus cells was not altered in basal control, IL-1β control, and IL-1β plus non-crosslinked HA groups. The inhibitory effects of HA3000 on IL-1β-stimulated MMP-1 and -3 expression were significantly reversed by pretreating tenocytes with OX-50 to inhibit CD44 expression. We found that HMW-HA (HA3000) attenuated MMP-1 and -3 expression in IL-1β-stimulated tenocytes by stimulating CD44 receptors.

This study has some limitations. First, we did not evaluate the expression of inflammatory cytokines or the downstream intracellular signaling pathways after HA-CD44 interaction. Additional studies on the comprehensive molecular mechanism are needed to better understand the function of HA in tendinopathy treatment. Second, we did not compare the different HA preparations by molecular weight in our clinical series. In clinical practice, the number of candidates who fit our criteria was small because it is rare to complete both the pretreatment and the posttreatment collection of LHB peritendinous effusion. The methodology that intervention with different commercial HA medications would lead to the inadequate case numbers in each subgroup and weaken the statistical power. Recent studies^15^,^19^ have reported the therapeutic effects of HMW-HA on tendinopathy; therefore, we used HMW-HA as our only treatment choice. Additional studies comparing commercial HA preparations with different molecular weight are necessary to confirm our results. Third, we examined only total protein levels of MMP-1 and -3, but that might not represent their real activity. Otherwise, the homeostasis among MMPs, endogenous tissue inhibitor of metalloproteinase protein (TIMP) and TIMP inhibitor might be altered after HA treatment. Further studies are necessary to clarify the dynamic changes and to examine the active form level of MMPs. Fourth, the functions of our candidate tendons in the *in vitro* model (Achilles tendon, an energy-storing tendon) and the *in vivo* model (LHB, a stabilizer tendon) are different. The difference might lead to an interpretation bias.

In this study, HA3000 significantly attenuated the mRNA and protein expression of MMP-1 and -3 in IL-1β-stimulated tenocytes. CD44 was clearly expressed in the plasma membranes of cultured tenocytes, but its expression level was not altered after various treatments. These effects of HA3000 were dose-dependent and were significantly reversed by pretreating tenocytes with OX-50 to inhibit CD44 expression. Clinically, HMW-HA treatment significantly reduced VAS scores and attenuated MMP-1 and -3 expression in patients with LHB tendinopathy. These findings indicated that HMW-HA might be an effective treatment for tendinopathy by attenuating MMP-1 and -3 expression. Additional studies on the mechanism of HA in tendinopathy are needed.

## Materials and Methods

### Ethics statement

All of the experimental rats were purchased from the Animal Center at National Cheng Kung University, and the following animal experiments were done strictly in accordance with protocols approved by the Institutional Animal Care and Use Committee of National Cheng Kung University (No. 101287). The human study was approved by the Institutional Review Board of National Cheng Kung University Hospital (No. ER-99–397), and was done strictly in accordance with the approved guidelines. Informed consent was obtained from all patients.

### Culturing and identifying tenocytes

Achilles tendons from 30 adult Sprague-Dawley rats (250–300 g) were harvested after the rats had been killed with an overdose of isoflurane. The paratendon sheath was removed. The endotendon samples were washed with sterile phosphate-buffered saline (PBS), minced into small pieces, and then cultivated in Dulbecco’s modified Eagle’s medium (DMEM) (Sigma-Aldrich, St. Louis, MO, USA) supplemented with 10% fetal calf serum and penicillin plus streptomycin (100 U/mL). The cells were maintained at 37 °C in a humidified incubator containing 5% CO_2_. The medium was changed every 3 to 4 days until confluent growth occurred. Well-characterized second-to-fourth passage cells were used in this experiment: they showed no phenotypic drift of major tenocyte markers such as cell shape (elongated and spindle shaped with apposition)^46^ and tenomodulin expression^47^. Cells were seeded (2 × 10^4^/well) on glass coverslips on a 3-cm dish cultured overnight, and then processed for immunoflourescent tenomodulin staining. A goat polyclonal anti-tenomodulin antibody (sc-49325; Santa Cruz Biotechnology, Santa Cruz, CA, USA) was used at concentrations of 1:100 in cultures with cells fixed in 1% paraformaldehyde in 0.1 M phosphate buffer (pH 7.4) for 5 min and then immediately washed in phosphate buffered saline (PBS). After they had been blocked with normal donkey serum at a concentration of 5% for 1 h, the cells were incubated with the primary antibody overnight. After additional washing and blocking, the secondary antibody donkey anti-gold IgG^+^ Alex 488 (Invitrogen, Carlsbad, CA, USA) was incubated for 1 h at room temperature. In parallel, the same concentration of the secondary antibody was used as the isotype control. DAPI (di amidino phenyl indole) fluorescent stain was used at a concentration of 1:2000 for 1 hour, after which the cells were observed under a fluorescent microscope (CKX31; Olympus Taiwan, Taichung).

### HA treatment in patients with long head of biceps tendinopathy

Consecutive patients with LHB tendinopathy were recruited from January 2012 to July 2013. Ten patients (3 men, 7 women; mean age: 62.6 years; age range: 49–72 years) were enrolled (Supplementary Table 1). The inclusion criteria were a chronic (>3 months) anterior shoulder pain and tenderness over the bicipital groove^26^ with LHB tendinopathic features and peritendinous effusion on ultrasound (US) imaging before and after HA treatments. The exclusion criteria were an LHB subluxation or dislocation or rupture, concomitant full-thickness rotator cuff tears, impingement syndrome, frozen shoulder syndrome, glenohumeral arthritis, connective tissue disease, or being < 18 years old^26^,^48^. The criteria for a diagnosis of LHB tendinopathy on US imaging were tendon enlargement and hypoechogenicity^28^. All patients were given three weekly US-guided LHB paratendinous injections of HA (Artiaid^®^Plus; Maxigen Biotech Inc., Taipei, Taiwan) (molecular weight: >1500 kDa; 15 mg/mL). The patient underwent the standard US shoulder examinations in sitting position. LHB tendinopathy with peritendinous effusion was confirmed using US imaging. The patient was next placed in supine position, and a sonographic transducer (SonoSite, 6–13 MHz; Advanced Technology Laboratories, Bothell, WA, USA), which was enclosed in a sterile surgical glove, was transversely positioned and remained at the bicipital groove. A 25-gauge, 1.5-inch needle was visualized in the long axis of the transducer and, with US guidance, was advanced using a lateral-to-medial approach. Once the needle tip was seen within the peritendinous effusion, the effusion was aspirated and collected for further analysis. HA (1 mL) was injected into the peritendinous area using real-time US monitoring. One month after the first HA injection, the LHB peritendinous effusion was confirmed, aspirated, and collected again. The intensity of the patient’s shoulder pain was recorded using a visual analog scale (VAS) score (0–100 points).

### IL-1β stimulation and HA treatments of various molecular weights

Tenocytes (3 × 10^5^) were seeded in 6-well plates and cultured in 2 mL of DMEM for 24 h. The serum-free DMEM was changed 1 h before drug treatment. The tenocytes were co-treated with rat recombinant IL-1β (10 ng/mL) (QZ2512081; R&D Systems, Minneapolis, MN, USA) and treated separately with three HA preparations of different molecular weight (HA350 kDa [HA350], HA1500 KDa [HA1500], and HA 3000 kDa [HA3000]) (2.5 mg/mL) for 24 h. The cells in the PBS group were the Control group and were not treated with IL-1β or HA. In the experiments involving CD44 blocking, the cells were pretreated with OX-50 (CBL1508; Millipore, Temecula, CA, USA) (10 μg/mL at 37 °C) for 30 min before the HA treatment^31^. The irrelevant rat IgG antibody (#02–9602, ThermoFisherScientific, Waltham, MA, USA) was used as the isotype control of OX-50. The previously described coverslip cultures were processed for immunofluorescent CD44 staining. OX-50 was used as the primary antibody at concentrations of 1:200 in cultures with cells fixed in 1% paraformaldehyde in 0.1 M phosphate buffer (pH 7.4) for 5 min and then immediately washed in phosphate buffered saline (PBS). After they had been blocked with normal donkey serum at a concentration of 5% for 1 h, the cells were incubated with OX-50 for 1 h. After additional washing and blocking, the secondary antibody goat anti-mouse IgG^+^ Alex 568 (Invitrogen, Carlsbad, CA, USA) was incubated for 1 hour at room temperature. In parallel, the same concentration of the secondary antibody was used as the isotype control. DAPI (di amidino phenyl indole) fluorescent stain was used at a concentration of 1:2000 for 1 h, after which the cells were observed using a confocal microscope (FluoView FV1000 MPE Multiphoton; Olympus Taiwan, Taichung). The various HA preparations were gifts from Maxigen Biotech Inc. (Taoyuan City, Taiwan).

### Proliferation and viability of tenocytes

A PrestoBlue assay (Invitrogen) was used to measure tenocyte *in vitro* cell proliferation. An MTT assay (Amresco, LLC, Solon, OH, USA) was used to evaluate *in vitro* cell viability in response to the various molecular weights of the HA preparations. After each group had been treated, the supernatant was removed from each well, and 20 μL of a 5-mg/mL MTT solution was added and incubated at 37 °C for 4 h. Formazan salt crystals were then dissolved with 150 μL of dimethylsulfoxide (DMSO) in each well. The mixtures were determined at 570 nm using a microplate reader. Dexamethasone (Dex), which toxic to tenocytes^41^, was used as a positive control in 10 nM and 100 nM treatment.

### Using reverse-transcription PCR and real-time PCR to measure MMP-1 and -3 mRNA expression

After 24-h of co-treatment^49^,^50^, the total RNA was extracted from the cultured cells using a reagent (Trizol; Invitrogen). Reverse-transcription PCR (RT-PCR) was done using cDNA and a kit (ReverTra Ace Set [PU-TRT-100]; Purigo Biotech Inc., Taipei, Taiwan). The reaction procedures consisted of incubation at 95 °C for 10 min, followed by 23 to 30cycles of 94 °C for 30 sec, 60 °C for 30 sec, 72 °C for 30 sec, and a final extension step at 72 °C for 10 min (Supplementary Table 2). Quantitative real-time RT-PCR (qRT-PCR) was done using SYBR Green dye and a real-time PCR System (StepOne Real-Time PCR System; Applied Biosystems [Life technologies], Rockville, MD, USA). After reverse transcription, qRT-PCR was done using the primers shown in Supplementary Table 3 under the following conditions: initial treatment at 95 °C for 10 min, denaturing at 95 °C for 15 sec, and annealing at 60 °C for 1 min without extension. This process was repeated for 40 cycles. The amounts of mRNA for MMP-1,MMP-3, and CD44 were measured and normalized against GAPDH as an internal standard based on the delta-delta-CT method.

### Using ELISA to measure MMP-1 and -3 mRNA

After 48-h of co-treatment^50^, cells were lysed and protein concentration was detected using the Bio-Rad method: 20 mg/mL of total MMP-1 and -3 mRNA protein was measured using specific ELISA kits ([rat: EIAab^®^, Wuhan, China] and [human: R&D Systems, Minneapolis, MN, USA]).

### HA preparations of various concentrations

After the above evaluations of the effects of HA solutions of various molecular weights, the effects on MMP-1 and -3 mRNA expression after treatment in different concentrations (0.1 mg/mL, 1 mg/mL, and 2.5 mg/mL) with the most optimal molecular weight HA were investigated using the above procedures.

### Statistical analysis

SPSS 16.0 for Windows was used for all data analysis. In the *in vitro* study, results are expressed as mean ± standard deviation. All data were normalized to GAPDH, an endogenous control. The parameters within *in vitro* groups were analyzed using Dunnett’s test. The correlation between various concentrations of HA and parameters were analyzed using the Spearman correlation rank test. In the *in vivo* study, the normality of data was analyzed using the Shapiro-Wilk test. Results are expressed as mean, median, and IQR because of the non-normal distribution of our data. The differences between parameters in the clinical group were compared using the Wilcoxon signed rank test. Significance was set at p < 0.05.

## Additional Information

**How to cite this article**: Wu, P.T. *et al*. High-molecular-weight hyaluronic acid attenuated matrix metalloproteinase-1 and -3 expression via CD44 in tendinopathy. *Sci. Rep.* **7**, 40840; doi: 10.1038/srep40840 (2017).

**Publisher's note:** Springer Nature remains neutral with regard to jurisdictional claims in published maps and institutional affiliations.

## Supplementary Material

Supplementary Tables
