# Supplementary material for: High-molecular-weight hyaluronic acid attenuated matrix metalloproteinase-1 and -3 expression via CD44 in tendinopathy
Source: Sci Rep. 2017 Jan 16;7:40840. doi: 10.1038/srep40840 (PMC5238506; doi:10.1038/srep40840)
Supplement: Supplementary Tables [file srep40840-s1.doc]

**High-molecular-weight hyaluronic acid attenuated matrix metalloproteinase-1 and -3 expression via CD44 in tendinopathy**

Po-Ting Wu^1,2,3,4^, Li-ChiehKuo^5^, Fong-Chin Su^3,4^, Shih-Yao Chen^6^, Tai-I Hsu^2,7^, Chung-Yi Li^8^,Kuen-Jer Tsai^9,10*^,I-Ming Jou^7,1,2*^

^1^Department of Orthopedics, College of Medicine, National Cheng Kung University, Tainan, Taiwan

^2^Department of Orthopedics, National Cheng Kung University Hospital, College of Medicine, National Cheng Kung University, Tainan, Taiwan

^3^Department of Biomedical Engineering, National Cheng Kung University, Tainan, Taiwan

^4^Medical Device Innovation Center, National Cheng Kung University, Tainan, Taiwan

^5^Department of Occupational Therapy, National Cheng Kung University, Tainan, Taiwan

^6^ Department of Internal Medicine, College of Medicine, National Cheng Kung University, Tainan, Taiwan

^7^Department of Orthopedics, E-DA Hospital, Kaohsiung, Taiwan

^8^Department of Public Health, College of Medicine, National Cheng Kung University, Tainan, Taiwan

^9^Institute of Clinical Medicine, College of Medicine, National Cheng Kung University, Tainan, Taiwan

^10^Center of Clinical Medicine, National Cheng Kung University Hospital, College of Medicine, National Cheng Kung University, Tainan, Taiwan

**Supplementary Table 1**. The demography data of ten patients with long head of biceps (LHB) tendinopathy before and after serial high-molecular weight hyaluronate (HMW-HA) treatment.

|  | Age |  | Symptom | VAS | | MMP-1 (pg/mL) | | MMP-3(pg/mL) | |
| --- | --- | --- | --- | --- | --- | --- | --- | --- | --- |
| Patient No. | (years) | Gender | Duration | Pretreatment | 1 Month | Pretreatment | 1 Month | Pretreatment | 1 Month |
|  |  |  | (months) |  |  |  |  |  |  |
| 1 | 49 | Male | 4 | 80 | 40 | 8301.4 | 1054.7 | 1508.1 | 847.2 |
| 2 | 72 | Female | 3.5 | 75 | 30 | 5929.5 | 2429.3 | 1256.1 | 1202.1 |
| 3 | 53 | Male | 6 | 85 | 40 | 5051.6 | 2864.4 | 1639.4 | 1488.8 |
| 4 | 65 | Female | 8 | 90 | 50 | 1850.9 | 665.9 | 821.4 | 552.4 |
| 5 | 55 | Female | 7 | 70 | 20 | 10566.0 | 2962.3 | 1529.9 | 1187.1 |
| 6 | 72 | Female | 8 | 80 | 20 | 11010.3 | 4300.2 | 589.9 | 490.9 |
| 7 | 63 | Female | 4 | 70 | 20 | 3069.0 | 1027.2 | 1923.0 | 1306.2 |
| 8 | 65 | Female | 5 | 70 | 30 | 35320.0 | 12959.0 | 1922.0 | 1036.8 |
| 9 | 70 | Male | 6 | 75 | 30 | 10006.0 | 2025.0 | 1104.0 | 1691.1 |
| 10 | 62 | Female | 5 | 80 | 10 | 1164.9 | 3524.3 | 1729.5 | 1922.7 |

Every patient received three weekly ultrasound-assisted LHB paratendinous injections of HA (Artiaid®Plus; Maxigen Biotech Inc., Taipei, Taiwan) (molecular weight: > 1500 kDa; 15 mg/mL).

1 Month: one month after initial HA treatment.

**Supplementary Table 2**. The sequences of target primers in reverse-transcription PCR

| **Gene Name** | **Sequences** |
| --- | --- |
| MMP-1(F) | 5´- ACAGTTTCCCCGTGTTTCAG -3´ |
| MMP-1(R) | 5´- CCCACACCTAGGTTTCCTCA -3´ |
| MMP-3(F) | 5´-TTCTCCAGGATCTCTGAAGGAGAGG-3´ |
| MMP-3(R) | 5´-ATTTGGTGGGTACCACGAGGACATC-3´ |
| CD44 (F) | 5´- TGGATCCGAATTAGCTGGAC -3´ |
| CD44 (R) | 5´- CCCGTTGAGTTCACTTGGTT -3´ |
| GAPDH(F) | 5´-TTCATTGACCTCAACTACAT-3´ |
| GAPDH(R) | 5´-GAGGGGCCATCCACAGTCTT-3´ |

PCR, polymerase chain reaction; MMP, matrix metalloproteinase; F, front; R, rear; GAPDH, glyceraldehyde-3-phosphate dehydrogenase.

**Supplementary Table 3**. The sequences of target primers in real-time PCR

| **Gene Name** | **Sequences** |
| --- | --- |
| MMP-1(F) | 5´-GCCAACAGGTGCAACAACAC-3´ |
| MMP-1(R) | 5´-GCATCAAGTTTACCTGGCAGATT-3´ |
| MMP-3(F) | 5´-TCCCAGGAAAATAGCTGAGAACTT-3´ |
| MMP-3(R) | 5´-GAAACCCAAATGCTTCAAAG ACA-3´ |
| CD44 (F) | 5´-AAGACATCGATGCCTCAAAC-3´ |
| CD44 (R) | 5´-CTCCAGTAGGCTGTGAAGTG-3´ |
| GAPDH(F) | 5´-GTATTGGGCGCCTGG TCA CC-3´ |
| GAPDH (R) | 5´-CGC TCC TGGAAGATG GTG ATG G-3´ |

PCR, polymerase chain reaction; MMP, matrix metalloproteinase; F, front; R, rear; GAPDH, glyceraldehyde-3-phosphate dehydrogenase.
